# Supplementary material for: Transcriptome Comparison Reveals Key Components of Nuptial Plumage Coloration in Crested Ibis
Source: Biomolecules. 2020 Jun 15;10(6):905. doi: 10.3390/biom10060905 (PMC7356354; doi:10.3390/biom10060905)
Supplement: Supplementary file 1 [file biomolecules-10-00905-s001.zip › supplementary files/Supporting_Information.docx]

| Sample^*^ | Concentration  (ng/μL) | Volume  (μL) | Total  (ng) | OD260/280 | OD260/230 | 28S/18S | RIN |
| --- | --- | --- | --- | --- | --- | --- | --- |
| WF1 | 205 | 52 | 10.66 | 2.03 | 2.21 | 2.1 | 9.9 |
| WF2 | 202 | 52 | 10.54 | 2.08 | 2.34 | 1.5 | 9.6 |
| BF1 | 152 | 32 | 4.86 | 1.80 | 2.04 | 1.3 | 8 |
| BF2 | 256 | 32 | 8.19 | 1.94 | 1.58 | 1.6 | 8.6 |

**Table S1. Summary of RNA quality of four samples used in the study**

*WF1: nonbreeding season ibis 1; WF2: nonbreeding season ibis 2; BF1: breeding season ibis 1; BF2: breeding season ibis 2.

**Table S2. Numbers of genes and actively expressed genes in the new gene set**

|  | All | Actively Expressed in One or More Samples（FPKM>1） |
| --- | --- | --- |
| All Protein-coding Genes | 19435 | 14808 |
| Known Protein-coding Genes | 17076 | 12753 |
| Novel Genes | 2359 | 2055 |

**Table S3. GO terms enriched in upregulated and downregulated DEGs of black skins on the neck of the breeding season ibises**

| Term type | GO accession | Description | DEGs | *q* |
| --- | --- | --- | --- | --- |
| Up | | | | |
| BP | GO:0009395 | phospholipid catabolic process | 4 | 3.44E-03 |
| MF | GO:0004620 | phospholipase activity | 5 | 8.06E-03 |
| MF | GO:0016298 | lipase activity | 5 | 1.12E-02 |
| BP | GO:0044242 | cellular lipid catabolic process | 4 | 1.14E-02 |
| Down | | | | |
| CC | GO:0005882 | intermediate filament | 33 | 4.82E-21 |
| CC | GO:0045111 | intermediate filament cytoskeleton | 33 | 4.82E-21 |
| MF | GO:0005200 | structural constituent of cytoskeleton | 22 | 3.25E-18 |
| CC | GO:0044430 | cytoskeletal part | 39 | 8.44E-11 |
| CC | GO:0005856 | cytoskeleton | 40 | 1.99E-10 |
| MF | GO:0005198 | structural molecule activity | 47 | 9.03E-09 |
| BP | GO:0051169 | nuclear transport | 17 | 5.04E-06 |
| BP | GO:0006606 | protein import into nucleus | 14 | 5.36E-05 |
| BP | GO:0034504 | protein localization to nucleus | 14 | 5.36E-05 |
| BP | GO:0044744 | protein targeting to nucleus | 14 | 5.36E-05 |
| BP | GO:0051170 | nuclear import | 14 | 5.36E-05 |
| BP | GO:1902593 | single-organism nuclear import | 14 | 5.36E-05 |
| BP | GO:0006913 | nucleocytoplasmic transport | 15 | 5.36E-05 |
| CC | GO:0043232 | intracellular non-membrane-bounded organelle | 44 | 8.16E-04 |
| BP | GO:0017038 | protein import | 14 | 9.42E-04 |
| CC | GO:0031012 | extracellular matrix | 16 | 1.27E-03 |
| CC | GO:0044421 | extracellular region part | 20 | 1.29E-03 |
| CC | GO:0043228 | non-membrane-bounded organelle | 44 | 1.41E-03 |
| CC | GO:0005581 | collagen trimer | 7 | 1.41E-03 |
| CC | GO:0042611 | MHC protein complex | 3 | 1.68E-03 |
| CC | GO:0042613 | MHC class II protein complex | 3 | 1.68E-03 |
| CC | GO:0005576 | extracellular region | 44 | 1.74E-03 |
| BP | GO:0022610 | biological adhesion | 23 | 2.06E-03 |
| MF | GO:0005509 | calcium ion binding | 28 | 2.09E-03 |
| MF | GO:0005201 | extracellular matrix structural constituent | 7 | 2.82E-03 |
| BP | GO:0007155 | cell adhesion | 22 | 3.51E-03 |
| CC | GO:0005578 | proteinaceous extracellular matrix | 11 | 5.58E-03 |
| BP | GO:0019882 | antigen processing and presentation | 4 | 1.40E-02 |
| BP | GO:0072594 | establishment of protein localization to organelle | 14 | 2.38E-02 |
| CC | GO:0043234 | protein complex | 59 | 2.44E-02 |
| BP | GO:0016482 | cytoplasmic transport | 16 | 2.63E-02 |
| MF | GO:0046872 | metal ion binding | 86 | 2.70E-02 |
| MF | GO:0043169 | cation binding | 86 | 3.17E-02 |
| MF | GO:0003993 | acid phosphatase activity | 12 | 4.80E-02 |
| MF | GO:0016638 | oxidoreductase activity, acting on the CH-NH2 group of donors | 5 | 4.80E-02 |

BP: biological process; CC: cellular component; MF: molecular function
